# PERCEPTIONS OF DEMENTIA FRIENDLY COMMUNITY STAKEHOLDERS IN THE US: CHALLENGES AND STRATEGIES DURING COVID-19

**DOI:** 10.1093/geroni/igac059.090

**Published:** 2022-12-20

**Authors:** Fei Sun, Ha-Neul Kim, Opur Fredrika, Christian Conyers

**Affiliations:** Michigan State University, East Lansing, Michigan, United States; Michigan State University, East Lansing, Michigan, United States; Michigan State University, East Lansing, Michigan, United States; University of Michigan, Ann Arbor, Michigan, United States

## Abstract

This study examines the challenges faced by Dementia Friendly Communities (DFC) during COVID-19 pandemic and the strategies used to address these challenges from the perspectives of DFC stakeholders in the U.S. Data were collected in 2020 through an online survey of 183 stakeholders (Mage =35.3, SD=8.8, 43.6% being female) involved who were in DFC design, implementation, or evaluation. Three challenges rated most critical by participants included limited funding (40.4%), difficulties to provide services due to policies to contain COVID-19 (35.7%), and lack of staffing (29.3%). Three rated most important strategies included seeking funding and government support (31.1%), developing partnerships and relationships with multiple sectors (29.1%), and recruiting persons with dementia and family caregivers as advocates (27.5%). Communities demonstrated resilience during the COVID-19 pandemic to implement DFC- related activities. To sustain DFC, enhancing national awareness, acquiring additional funding, and firm cogency from staff members, local/state government, and local communities are needed.

